# Diversity, Distribution and Co-occurrence Patterns of Bacterial Communities in a Karst Cave System

**DOI:** 10.3389/fmicb.2019.01726

**Published:** 2019-08-06

**Authors:** Hai-Zhen Zhu, Zhi-Feng Zhang, Nan Zhou, Cheng-Ying Jiang, Bao-Jun Wang, Lei Cai, Shuang-Jiang Liu

**Affiliations:** ^1^State Key Laboratory of Microbial Resources and Environmental Microbiology Research Center at Institute of Microbiology, Chinese Academy of Sciences, Beijing, China; ^2^College of Life Sciences, University of Chinese Academy of Sciences, Beijing, China; ^3^State Key Laboratory of Mycology at Institute of Microbiology, Chinese Academy of Sciences, Beijing, China; ^4^Research Center for Eco-Envorinmental Sciences-Institute of Microbiology, Chinese Academy of Sciences-University of Chinese Academy of Sciences, Joint-Lab of Microbial Technology for Environmental Science, Beijing, China; ^5^College of Resources and Environment, University of Chinese Academy of Sciences, Beijing, China

**Keywords:** karst cave, bacteriomes, community composition and abundance, bacterial diversity, co-occurrence pattern

## Abstract

Caves are typified by their permanent darkness and a shortage of nutrients. Consequently, bacteria play an important role in sustaining such subsurface ecosystems by dominating primary production and fueling biogeochemical cycles. China has one of the world’s largest areas of karst topography in the Yunnan-Guizhou Plateau, yet the bacteriomes in these karst caves remain unexplored. In this study, bacteriomes of eight karst caves in southwest China were examined, and co-occurrence networks of cave bacterial communities were constructed. Results revealed abundant and diversified bacterial communities in karst caves, with *Proteobacteria*, *Actinobacteria*, and *Firmicutes* being the most abundant phyla. Statistical analysis revealed no significant difference in bacteriomes among the eight caves. However, a PCoA plot did show that the bacterial communities of 128 cave samples clustered into groups corresponding to sampling types (air, water, rock, and sediment). These results suggest that the distribution of bacterial communities is driven more by sample types than the separate caves from which samples were collected. Further community-level composition analysis indicated that *Proteobacteria* were most dominant in water and air samples, while *Actinobacteria* dominated the sediment and rock samples. Co-occurrence analysis revealed highly modularized assembly patterns of the cave bacterial community, with *Nitrosococcaceae* wb1-P19, an uncultured group in *Rokubacteriales*, and an uncultured group in *Gaiellales*, being the top-three keystone members. These results not only expand our understanding of cave bacteriomes but also inspires functional exploration of bacterial strains in karst caves.

## Introduction

Karst is a topography formed by the dissolution of soluble rocks such as limestone, dolomite, and gypsum, and is characterized by underground drainage systems featuring sinkholes and caves. Karst landforms are widespread; approximately 20% of the earth’s dry ice-free surface is composed of karst terrain (Ford and Williams, 1989; Legatzki et al., 2011). Caves are one of the typical features of a subsurface karst, developing when acidic water starts to break down the bedrock near cracks. More than 50,000 cave systems have been identified in the United States (Barton and Jurado, 2007), while China hosts a large contiguous karst landscape of ca. 550,000 km^2^ in extent, and seven karst clusters in south China are listed as a World Heritage Property by UNESCO. This region is recognized as the world’s foremost area for karst landform development in the humid tropics and subtropics^1^. In particular, karst caves are mostly distributed in the southwest of the Yunnan-Guizhou plateau, of which the longest exceeds 138 km (Zhang and Zhu, 2012).

Because caves are characterized by darkness, low to moderate temperatures, high humidity, and limited nutrients, they can be discriminated from land surface substrates (Gabriel and Northup, 2013). Despite their oligotrophic conditions, microbial communities thrive in caves, with the average number of microorganisms growing in these ecosystems reaching 10^6^ cells/g of rock (Barton and Jurado, 2007). In the absence of sunlight, microorganisms in cave habitats cannot photosynthesize, and are forced to rely on alternative primary production strategies to offset the lack of an exogenous carbon source. Chemoautotrophic bacteria in a Romanian cave were found capable of fixing inorganic carbon and using hydrogen sulfide as an energy source, and their chemoautotrophic production also supported cave-adapted invertebrates (Sarbu et al., 1996). Geobiological evidence in recent years suggests that subterranean karst environments might be a global sink for atmospheric methane due to processes involving microbes (Nguyễn-Thuý et al., 2017; Webster et al., 2018; Zhao et al., 2018). A better understanding of the microbial community of cave ecosystems will not only expand our knowledge of global microbial diversity, but it also provides valuable information about energy dynamics of novel community assemblages.

Early studies of cave microbiology mostly involved traditional cultivation techniques, and those bacteria strains playing a role in limestone calcification and primary nutrient production were identified (Danielli and Edington, 1983; Cunningham et al., 1995; Baskar, 2014). Attention was given in particular to cave *Actinobacteria*, which were regarded as a potential source of novel bioactive compounds (Axenovgibanov et al., 2016; Ghosh et al., 2017; Adam et al., 2018). The advent of new tools for the study and characterization of microbiomes greatly facilitated investigations of cave microbial community structure and functionality, rapidly advancing our knowledge of cave ecosystems (Barton et al., 2004; Ortiz et al., 2013; Wu et al., 2015). Previous investigations of microbial communities mostly focused on selected samples, such as its dripping waters (Laiz et al., 1999; Liu et al., 2010; Marques et al., 2019), cave sediments (Adetutu et al., 2012), cave wall surfaces (Pasić et al., 2010; Ortiz et al., 2013) and biofilms (Jones et al., 2010, 2012, 2014), and on how factors such as pH (Yun et al., 2016a), nutrition (Cloutier et al., 2017), and trace elements (Wu et al., 2015) could shape microbial communities in caves. Yet, systematic profiling of the cave bacteriomes sourced from air, water, rock, and sediment samples has not been reported.

It is estimated that China has hundreds of thousands of caves, but studies on bacterial communities have mainly focused on two caves, i.e., a ∼250 m long karst cave in central China (Liu et al., 2010; Yun et al., 2016a, b; Zhao et al., 2018) and a limestone cave in the western Loess Plateau (Wu et al., 2015). Recently, samples of air, water, rock, and sediment were intensively collected from eight karst caves in southwest China. The study of their mycobiomes indicated that both sample types and spatial variables were the key determinants of fungal distributions (Zhang and Cai, 2019). In this report, we focus on the features of and factors shaping the bacterial community in those same eight caves. We hypothesize that microbial communities of air, water, rock and sediment are shaped by their specific niche traits and that there are indicator microbial groups in each niche.

## Materials and Methods

### Sample Collection and Property Measurement

Caves investigated in this study are located in southwest China; namely, in the Chongqing, Sichuan and Yunnan provinces, and the Guangxi Zhuang autonomous region (Supplementary Figure 1 and Supplementary Table 1). The geographic sampling area ranged from ca. 23° to 30° in latitude and from ca. 102° to 110° in longitude, with a subtropical monsoon climate. Limestone strata are abundant in the area, having a total deposit thickness of more than 10,000 m (Zhang and Zhu, 2012), readily promoting a karst landscape with the corrosion of surface and ground water.

The cave sampling strategy was described in our previous study (Zhang and Cai, 2019). In each cave, four to six sampling sites, interspersed at equal distances, were chosen along a transect. At each site, 2 m^3^ of air was filtered through a 0.22 μm sterile polyester membrane using an air sampler (MAS-100Eco, Merck Millipore, Darmstadt, Germany) at a velocity of 100 L/min for 20 min; 2 L of water was filtered through a sterile 0.22 μm polyester membrane; 30 g of sediment was collected using an autoclaved spade at shallow depth (1.0–5.0 cm) after removing the surface layer (c. 1.0 cm) from three different spots; five pieces of rock in different orientations were sampled with an autoclaved chisel inserted into existing cracks, followed by hammer blows (Ruibal et al., 2005). All the samples were kept cold and transferred in sterile zip-lock plastic bags at 4°C, and then stored at −80°C before further processing.

Temperature and air humidity of each sampling site was measured, *in situ*, with a hydro thermograph (HT-853, HCJYET, Beijing, China). The pH value of sediment and rock samples was measured with a pH meter (FE20, Mettler Toledo, Zurich, Switzerland), for which the samples were dried, ground, and fully mixed with distilled water in a ratio of 1:2.5 (w/v) before this measurement (Sparks et al., 1996). The moisture of sediment samples was calculated after drying them at 105°C to a constant weight (Chen, 2003). Total organic carbon (TOC) and total nitrogen (TN) were measured using a TOC/TN analyzer (Vario TOC, Elementar, Hanau, Germany).

### DNA Extraction From Samples

Samples on a polyester membrane were washed off with sterile water and collected by centrifugation. The surface of rock samples was washed with 95% ethanol to reduce the contaminating influence of any dust and airborne spores, followed by two washes with sterile water containing 0.1% of the weak detergent Tween 20 (Ruibal et al., 2005). Pre-processed rock samples were then pulverized with a sterile mortar for DNA extractions.

DNA was extracted using the FastDNA^®^ Spin Kit (MP Biomedicals, Solon, OH, United States), following the manufacturer’s instructions, and the concentration and purity of the extracted DNA was measured by a NanoDrop 2000 spectrometer (Thermo Fisher Scientific, Wilmington, DE, United States). V4 variable regions of the 16S rRNA gene were amplified using two universal primers, 515F (5′-GTGCCAGCMGCCGCGGTAA-3′) and 806R (5′-GGACTACHVHHHTWTCTAAT-3′) (Caporaso et al., 2011), with barcodes at the 5′ end of the forward primer. The PCR reaction was performed in 50 μl volume, containing 1.5 μl of both primers (10 μmol/L), 25 μl of 2× KAPA HiFi HotStart ReadyMix (Kapa Biosystems, MA, United States) and 23 μl of template DNA. The PCR procedure included an initial denaturation at 95°C for 3 min, 32 cycles of denaturing (20 s at 98°C), annealing (16 s at 54°C), and extension (16 s at 72°C), and a final extension at 72°C for 1 min. Triplicate PCR products of each sample were pooled and purified using AMPure XP beads (Beckman Coulter, IN, United States). The pooled products were quantified using the Qubit dsDNA HS Assay Kit (Invitrogen, CA, United States), and then diluted to reach an equal concentration. The libraries were indexed using the NEB Next Ultra DNA Library Prep Kit for Illumina (New England Biolabs, United States), and finally sequenced on an Illumina MiSeq PE 250 machine with the MiSeq Reagent Kit v2 (500 cycles; 2 × 250 bp).

### 16S rRNA Gene Amplicon Analysis

Sequences were demultiplexed according to their barcodes. The adapter, primer, and barcode were each subsequently removed. Paired-end sequences were merged using PEAR software (v0.9.6) with the *p*-value set at 0.01 (Zhang et al., 2014). Quality control (fastq_maxee = 0.5), replication, singleton and chimera removal, and OTU (operational taxonomic unit) clustering (at 97% similarity) were performed using USEARCH v7.0.1090 (Edgar, 2010). The corresponding OTU table, providing the abundances of bacterial taxa in different samples, was made with QIIME v1.9.0 (Caporaso et al., 2010) using the SILVA database release 132 (Pruesse et al., 2012; Quast et al., 2013; Yilmaz et al., 2014). Mitochondrial and chloroplastic DNA sequences, as well as OTUs with a total relative abundance of <0.00001 in all samples were discarded using customized R scripts. For the alpha diversity (the species richness and evenness) assessment, sequences in each sample were rarefied into the same number, and for the beta diversity (the dissimilarity among samples in terms of community composition) assessment, the OTU table was normalized using the CSS method (Paulson et al., 2013).

### Statistical Analysis and Visualization

All the statistical analyses of data were performed in the R platform v3.4.2^2^ with the use of its packages. Normal distributions of the data were checked with the Shapiro–Wilk test and homoscedasticity of variances was analyzed using Bartlett’s test. Significant differences in the variance of parameters were evaluated with ANOVA (analysis of variance) or Kruskal–Wallis tests; their corresponding *post hoc* comparisons were made using Tukey’s HSD (honestly significant difference) test in the “stats” package or Dunn’s multiple comparison test in the “FSA” package^3^.

Visualization of diversity and distribution of cave-associated bacterial communities were performed using the “ggplot2” package unless otherwise stated (Wickham, 2009). A map indicating the location of the eight sampled caves was plotted using the “maptools” package for R (Lewin-Koh et al., 2011). Principal coordinates analysis (PCoA) was conducted using the cmdscale function in the “vegan” package^4^. To statistically support the visual clustering of bacterial communities in the PCoA analysis, bacteriomes in different cave niches were compared using permutation-based hypothesis tests (PERMANOVA), with *n* = 999 random permutations. Spearman correlations between the Shannon index and cave environmental factors was calculated using the corr. test function in the “psych” package (Revelle, 2018). The Bray–Curtis dissimilarity of cave bacterial communities was fitted with environmental factors using the envfit function in the “vegan” package. The Venn diagram was drawn using the “VennDiagram” package (Chen and Boutros, 2011). Indicators of different caves niches were predicted using the multipatt function in the “indicspecies” package, with *n* = 999 random permutations (Cáceres and Legendre, 2009; Cáceres et al., 2010, 2011, 2012). Beta diversity partitioning was performed using the “betapart” package, which partitioned the diversity into component richness and species turnover (Baselga and Orme, 2012). The comparison between OTUs in air samples and the other three types of samples was conducted using “edgeR” package (Robinson et al., 2010).

To reduce network complexity, those genera with relative abundances >0.05% in both the whole dataset and in each cave niche were selected to build co-occurrence networks (Jiao et al., 2016; He et al., 2017). Spearman’s correlation coefficient between two genera was considered statistically robust if its value (ρ) was >0.6 with a corresponding *p*-value of <0.01 (Barberán et al., 2011). The pairwise comparisons based on genera abundance were performed using the rcorr function in the “Hmisc” package^5^, and the *p*-values adjusted using the “fdrtool” package (Strimmer, 2008a, b). Co-occurrence networks, with each node representing one genus and each edge denoting a strong and significant correlation, were built using the “igraph” package; visualization of networks and calculation of network topological properties were performed using the interactive platform Gephi (Newman, 2003; Csardi and Nepusz, 2006; Newman, 2006; Bastian et al., 2009).

## Results

### Geochemical Characteristics of the Eight Karst Caves

The major geographical and physicochemical characteristics of sampled caves are listed in Table 1. The base rock of these caves was mostly limestone and/or dolomite. Though local residents and cave explorers occasionally entered these caves, all eight have never been accessible for massive tourist visits, and so remains pristine to a great extent. Seven of the eight sampled caves are located in mountains covered by forest, and another one (cave S7) is located in farmland area with tobacco seedlings. The average annual precipitation in the area of each cave ranged from 900 mm (cave Y2) to 1900 mm (cave G1). The temperature while sampling the caves ranged from 11.2 to 25.1°C, reflecting a combined location variation owing to latitude as well as elevation. Caves G1 and G3 are at relatively low latitude and elevation, and their temperature was the highest among the eight caves. Nevertheless, all the caves had high humidity (80–91%) and were alkaline (pH 7.9–8.3), in accordance with the nature of their base rock. No significant differences were found in the pH levels among the sampled caves.

**TABLE 1:** Environmental characteristics of the eight sampled karst caves.

	**Caves**							
**Environmental factors**	**C1**	**C2**	**G1**	**G3**	**S7**	**S8**	**Y2**	**Y3**
Latitude (^*o*^N)	29°35′06.09′′	29°35′27′′	24°56′30.55″	23°24′36″	28°11′24.6″	30°24′36″	25°08′04″	24°28′11.52′
Longitude (^*o*^E)	108°00′02.28″	108°00′02″	110°30′37.93″	108°55′52″	105°08′21.6″	106°52′42″	103°22′57″	102°50′31.8′
Elevation (m)	890	1020	269	129	750	850	1870	1840
Provence	Chongqing	Chongqing	Guangxi	Guangxi	Sichuan	Sichuan	Yunnan	Yunnan
Temperature (^*o*^C)^∗^	11.8 ± 0.7^c^	11.2 ± 0.3^c^	25.1 ± 1.1^a^	25.0 ± 0.0^a^	12.5 ± 1.5^c^	14.2 ± 0.4^bc^	15.7 ± 1.1^b^	16.5 ± 1.1^ab^
Air humidity (%)^∗^	91 ± 2^a^	91 ± 2^a^	87 ± 4^b^	80 ± 0^c^	91 ± 1^a^	91 ± 1^a^	85 ± 7^b^	84 ± 3^b^
pH^∗^	8.2 ± 0.5^a^	8.2 ± 0.5^a^	8.0 ± 0.4^a^	8.2 ± 0.5^a^	7.9 ± 0.4^a^	8.3 ± 0.6^a^	8.1 ± 1.1^a^	8.1 ± 0.7^a^

**^∗^Data are shown as the mean ± standard deviation. Means labeled with different superscript lettering (a, b, or c) indicate significant differences among caves. Statistical significances were determined by the Kruskal–Wallis test, followed by Dunn’s multiple comparisons, at p < 0.05.**

### Distribution and Abundance of Bacterial Taxa in Caves

**FIGURE 1:**
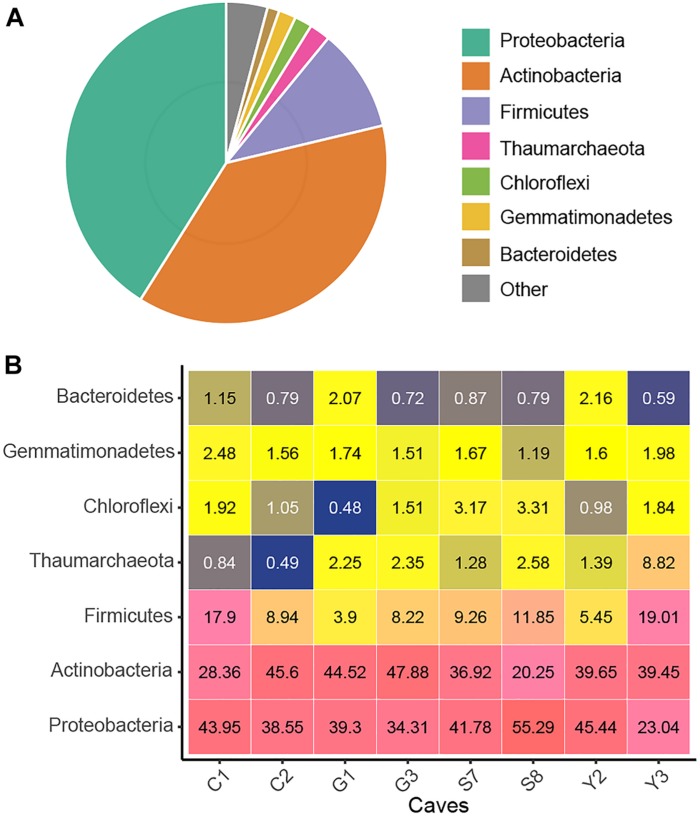
Taxonomic composition of the karst cave bacterial community. Pie chart showing the average relative abundances of all detected phylum in the whole dataset **(A)**. Heatmap showing the average relative abundances (%) of the major cave bacterial phyla in each sampled cave **(B)**; the number within each box represents the median abundance of the taxa in a given cave, taxa with low abundance are colored blue, those in higher abundance are yellow, while the highest are in pink.

After quality control and removal of mitochondria and chloroplast sequences, a data set of 16S rRNA genes (V4 region) containing 4,772,351 clean sequences was obtained. The number of sequences from each sample ranged from 24,093 to 51,945, with an average of 37,683. These sequences were clustered into 11,444 OTUs based on a 97% similarity threshold, of which 97.8% were assigned bacterial phyla (Supplementary Table 2). *Proteobacteria*, *Actinobacteria*, and *Firmicutes* were predominant (i.e., relative abundance >10%) in cave microbial communities, accounting for 89.1% of the total sequences. The *Proteobacteria* (relative abundance = 41.1%) were represented by the classes *Gammaproteobacteria* (32.4%) and *Alphaproteobacteria* (7.9%). The other two dominant phyla, *Actinobacteria* (37.6%) and *Firmicutes* (10.4%), were represented, respectively, by *Actinobacteria* (25.2%) and *Bacilli* (9.6%) at the class levels Additionally, the archaeal phylum *Thaumarchaeota* as well as the bacterial phyla *Gemmatimonadetes*, *Chloroflexi*, and *Bacteroidetes* were also detected at relative abundances of >1% in the cave communities (Figure 1). At a finer taxonomic level, 21 taxa at the family level each had an average relative abundance of more than 1%, among which *Pseudonocardiaceae* and *Enterobacteriaceae* were remarkably abundant (Supplementary Figure 2).

### Assembly Patterns of the Cave Bacterial Community

Alpha diversity of cave bacteriomes was measured by Shannon and Chao 1 indices. There was no significant difference in alpha diversities among the eight caves (Shannon: ANOVA, *p* > 0.05; Chao 1: Kruskal–Wallis test, *p* > 0.05, Supplementary Figure 3). However, the Chao 1 indices of sediment and water samples were significantly higher than those of rock and air samples, according to the Kruskal–Wallis test followed by Dunn’s multiple comparisons (*p* < 0.05). Shannon indices confirmed that sediment samples had a significantly higher diversity than the rock and air samples did, but no significant difference was observed between water samples and the other three types of samples (Table 2).

**TABLE 2:** Characteristics of cave bacteria richness and diversity indices among different karst cave niches.

**Cave niche**	**Observed OTUs**	**Coverage (%)**	**Chao 1**	**Shannon’s index**
Air	1161 ± 429^b^	98.4 ± 0.8^a^	1583 ± 640^b^	6.50 ± 1.25^b^
Rock	1129 ± 351^b^	98.4 ± 0.6^a^	1617 ± 528^b^	7.03 ± 0.83^b^
Sediment	1449 ± 556^a^	97.9 ± 0.9^b^	2051 ± 776^a^	7.69 ± 1.13^a^
Water	1484 ± 711^ab^	97.6 ± 1.1^b^	2207 ± 1005^a^	7.07 ± 1.51^ab^

**Data are shown as the mean ± standard deviation. Means labeled with different superscript lettering (a and b) indicate significant differences between cave niches for a given index. Statistical significances were determined by the Kruskal—Wallis test, followed by Dunn’s multiple comparisons, at p < 0.05.**

**FIGURE 2:**
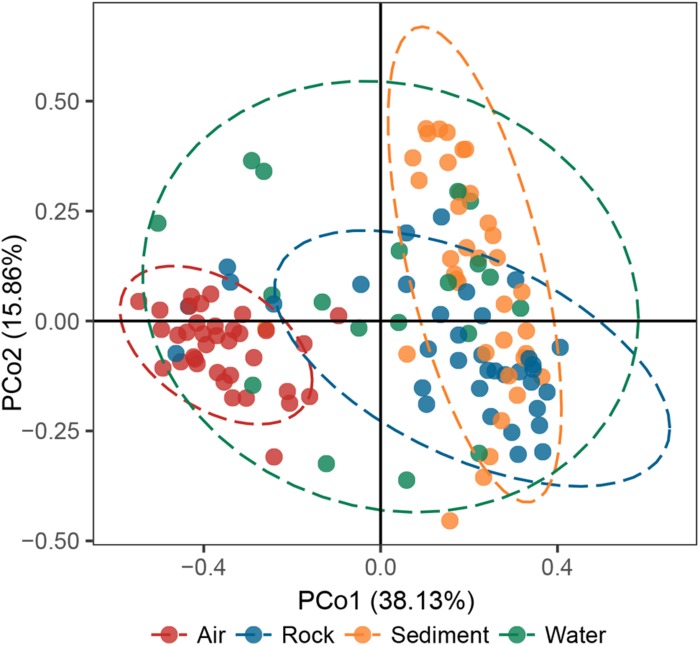
Principal coordinates analysis (PCoA) ordination of variation based on weighted UniFrac dissimilarity. The dotted lines indicate the 95% confidence intervals grouped by cave niche.

The principal coordinates analysis (PCoA) plot revealed that bacterial communities in cave samples generally clustered in line with their cave niches (Figure 2). Members of air samples were closely clustered and clearly separated from samples of other types, while members of water samples were loosely clustered. These results indicated that as the medium for the assembly of bacterial communities, cave niches played a key role in determining cave bacterial diversity. This observation is reasonable, because different cave niches differ in their nutrient levels. Moreover, different niches also represent unique origin models as growing substrates for bacterial communities: rocks are cave-originating, with limited nutrients from water leakage or condensation of water moisture from surroundings; sediments are brought into caves from the outside environment and retained in caves, however, water and air are more apt to undergo frequent exchanges with the outside environment, as well as other niches inside the cave, due to their flow.

To disclose the driving force of bacterial community assembly, the possible influence of cave environmental factors (Supplementary Table 3) on alpha and beta diversity was evaluated (Table 3). It was observed that temperature and air humidity significantly differentiated bacterial communities but had no significant effects on species diversity, when pooling all samples from the eight caves. But when each type of sample was analyzed separately, three results emerged: (1) Humidity showed a significant and negative correlation with the Shannon indices, while temperature and humidity statistically affected community composition, for air samples. (2) No factors tested were strongly related to Shannon indices of rock or water samples, yet TOC took a significant part in differentiating bacterial communities in their respective samples. (3) Temperature, air humidity, TOC, TN, and moisture all significantly contributed to bacterial community differentiation in sediment samples, with moisture and TN having positive and negative correlations with sediment’s Shannon indices.

**TABLE 3:** Analysis of the effect of environmental factors on karst cave bacterial diversity.

		**Sum**		**Air**		**Rock**		**Sediment**		**Water**	
	**Environmental factors**	**ρ**	***p***	**ρ**	***p***	**ρ**	***p***	**ρ**	***p***	**ρ**	***p***
Shannon index	Depth^a^	−0.1254	0.158	−0.3242	0.054	0.1567	0.361	−0.1753	0.299	−0.2115	0.385
	Temperature	0.0655	0.463	0.2868	0.090	0.1506	0.381	−0.1268	0.455	−0.0850	0.729
	Air humidity	−0.0424	0.635	**−0.3641**	**0.029^∗^**	−0.1344	0.435	0.2324	0.166	0.2598	0.283
	pH	–	–	–	–	0.04725	0.784	−0.0702	0.684	–0.0490	0.852
	TOC^b^	–	–	–	–	−0.0263	0.879	−0.1390	0.419	–0.2010	0.439
	TN^c^	–	–	–	–	−0.0577	0.738	**−0.5122**	**0.001^∗∗∗^**	–0.0343	0.896
	Moisture	–	–	–	–	–	–	**0.5197**	**0.001^∗∗∗^**	–	–

	**Environmental factors**	***R*^2^**	***p***	***R*^2^**	***p***	***R*^2^**	***p***	***R*^2^**	***p***	***R*^2^**	***p***

Bray-curtis dissimilarity	Depth^a^	0.0058	0.783	0.0707	0.293	0.0376	0.513	0.0568	0.384	0.1459	0.333
	Temperature	**0.1052**	**0.015^∗^**	**0.3777**	**0.001^∗∗∗^**	0.1220	0.120	**0.2180**	**0.015^∗^**	0.2091	0.185
	Air humidity	**0.1446**	**0.002^∗∗^**	**0.3022**	**0.008^∗∗^**	0.1680	0.067	**0.2312**	**0.013^∗^**	0.3556	0.056
	pH	–	–	–	–	0.0754	0.274	0.0298	0.583	0.2682	0.113
	TOC^b^	–	–	–	–	**0.2199**	**0.032^∗^**	**0.1704**	**0.045^∗^**	**0.5639**	**0.003^∗∗^**
	TN^c^	–	–	–	–	0.1009	0.183	**0.2673**	**0.016^∗^**	0.0859	0.563
	Moisture	–	–	–	–	–	–	**0.4577**	**0.001^∗∗∗^**	–	–

**^*a*^Refers to the distance between cave entrance and sampling site. ^*b*^Total organic carbon. ^*c*^Total nitrogen. –, not applicable; ^∗^*p* < 0.05; ^∗∗^*p* < 0.01; ^∗∗∗^*p* ≤ 0.001; ρ, Spearman’s correlation coefficient; R^2^, goodness of fit; the values associated to statistically effective factors are shown in bold.**

### Differences and Indicator Taxa of Microbial Community Among Cave Niches

**FIGURE 3:**
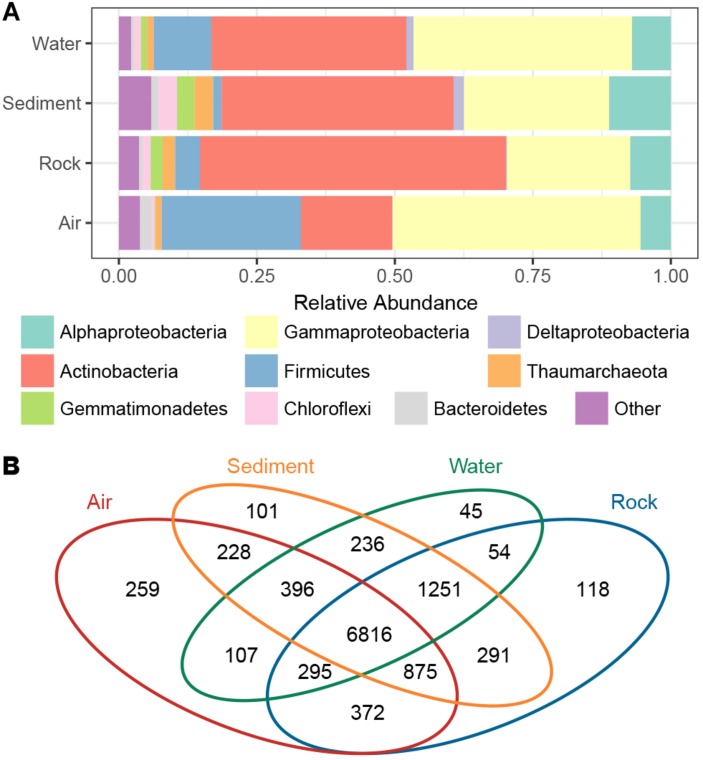
The proportion of bacterial phyla/subphyla in the four cave niches **(A)**. Venn diagram of the exclusive and shared OTUs found among the different cave niches **(B)**.

The taxonomic composition of bacterial groups in the four cave niches (air, water, rock, and sediment) was further evaluated (Figure 3A). *Proteobacteria* was the most dominant phylum in water and air samples (47.9 and 50.5%, respectively), while *Actinobacteria* was the most dominant in sediment and rock samples (42.1 and 55.4%, respectively). *Firmicutes* in air samples (25.3%) accounted for a larger proportion of bacteria found there compared to the other three cave niches (water, rock, sediment). When the *Proteobacteria* in each cave niche was analyzed at the class level, *Gammaproteobacteria* was overwhelmingly dominant in air and water samples (44.9 and 39.6%, respectively), *Deltaproteobacteria* were more abundant in water and sediment samples than in air and rock samples, and *Alphaproteobacteria* was more abundant in sediment than in the other three niches.

The Venn diagram revealed that OTUs differed among the four cave niches (Figure 3B). In accordance with the PCoA plot distributions, the most-clustered air samples had the greatest number of niche specific OTUs while the least-clustered water samples had the fewest number of niche-specific OTUs. A total of 6,816 OTUs were shared among all four cave niches. Although more than half of the total OTUs were present in each niche, when partitioning beta diversity into component richness and turnover, the latter accounted for the bulk of the difference found.

**FIGURE 4:**
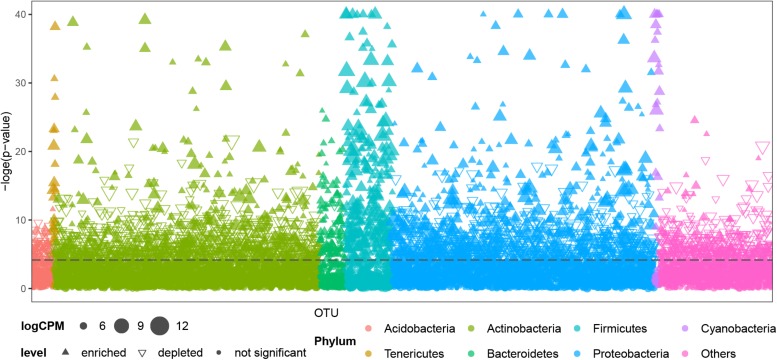
Comparison of OTUs’ distribution between air samples and other samples (niches) from caves. CPM is counts per million, the dashed line is the 95% confidence level. Solid upward triangles are OTUs significantly enriched in air samples, hollow downward triangles are OTUs significantly depleted in air samples, solid points are those OTUs significantly unchanged in air samples compared with other types of samples. The size of triangles and points are proportional to the abundance of that OTU.

Air samples in cave systems are rarely studied, and they harbor unique prokaryotic communities that differ significantly from the other three, more intensively studied types of samples (PERMANOVA, *p* < 0.01, Supplementary Table 4). Comparisons were then made between OTUs in air samples and other cave niches to better understand the distinctiveness of cave air’s bacteriome. Most OTUs belonging to *Tenericutes*, *Bacteroidetes*, *Firmicutes*, and *Cyanobacteria* were significantly enriched in air samples; a few OTUs in *Proteobacteria* and *Actinobacteria* were significantly enriched in terms of relative abundance while others were depleted; most OTUs in *Acidobacteria* and other phyla were significantly depleted in air samples (Figure 4).

**FIGURE 5:**
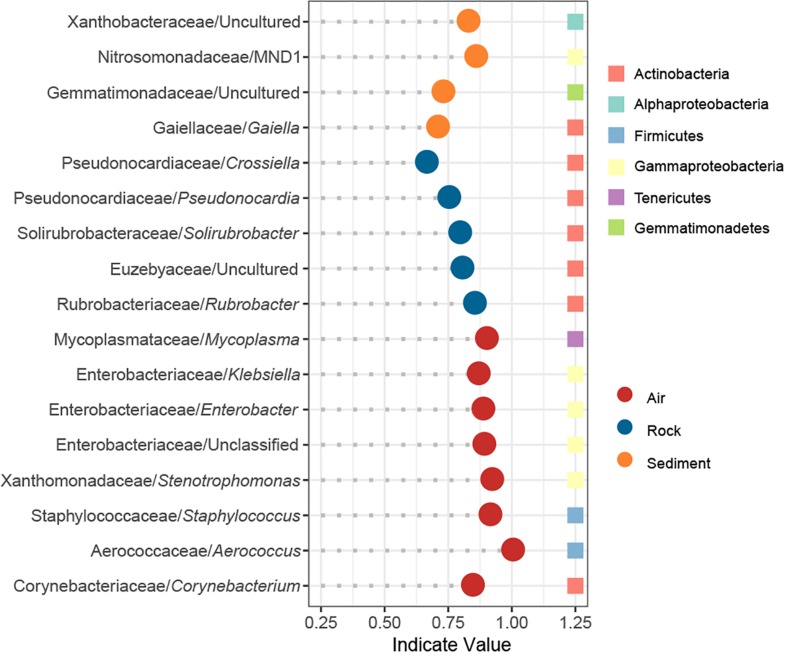
Indicator genera of different cave niches based on an IndVal analysis. Genera with an indicator value >0.6 and *p* < 0.05 were identified as indicators.

The IndVal analysis detected a total of 17 indicator genera that were significantly associated with different cave niches (Figure 5). In accordance with the numbers of niche specific OTUs in the Venn diagram, water samples showed no indicator genus whereas air samples had as many as eight indicator genera. Rock samples and sediment samples overlapped considerably in the PCoA plot, and their composition patterns at the phylum level were relatively similar, yet there were still five and four indicator genera that reflected their distinctive features, respectively. Taxonomically, all indicators in rock samples belonged to *Actinobacteria*, and half of the indicators in air samples were *Gammaproteobacteria*.

### Co-occurrence Patterns of Bacterial Communities in the Cave Environment

**FIGURE 6:**
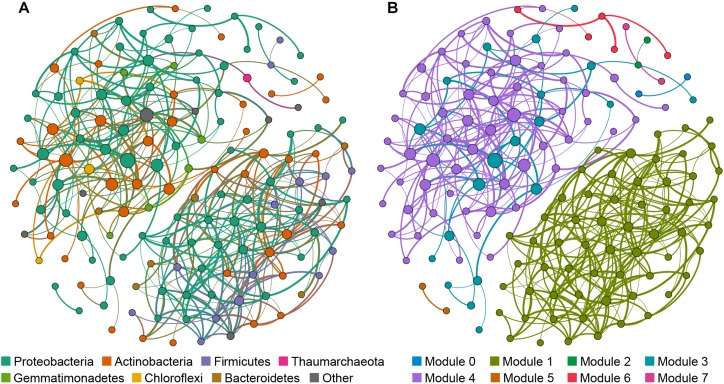
Network of co-occurring bacterial genera based on a correlation analysis for the cave environment. A connection denotes a strong (Spearman’s ρ > 0.6) and significant (*p* < 0.01) correlation. The nodes in network **(A)** are colored according to phylum, while the nodes in network **(B)** are colored with respect to modularity class. Node size is proportional to the betweenness centrality of each genus, and edge thickness is proportional to the weight of each correlation.

The cave bacterial network was generated for cave samples and consisted of 150 nodes (genera) and 399 edges (Figure 6). Topological properties were calculated to describe the complex pattern of inter-relationships among nodes: The average path length (APL) was 2.856 edges with a network diameter (ND) of eight edges, the clustering coefficient (CC) was 0.107 and the modularity index (MD) was 0.549. The nodes in the network were assigned to 12 bacterial phyla, among which three phyla (*Proteobacteria*, *Actinobacteria*, *Firmicutes*) were widely distributed, accounting for more than 83% of all nodes (Figure 6A). Based on the betweenness centrality (BC) scores, the top-three genera identified as keystone taxa were *Nitrosococcaceae* wb1-P19, an uncultured group in *Rokubacteriales*, and an uncultured group in *Gaiellales*.

Modules are densely-linked network regions that have more links inside than outside (Bissett et al., 2013). When the nodes were modularized, most nodes grouped into four major modules (Figure 6B), whose respective nodes showed different preferences to cave niches. For example, the majority of nodes in module 1 were the most abundant in air samples, the majority of nodes in module 4 were the most abundant in sediment samples, while nodes in module 3 were most abundant in rock or sediment samples, and nodes in module 6 were most abundant in water samples (Supplementary Table 5). This meant that bacterial communities in each niche of the caves could have had more interactions within the niche instead of outside it.

To further illustrate the bacteria’s co-occurrence patterns within each cave niche, bacterial networks were constructed separately for these four types of samples (Supplementary Figure 4), and their corresponding topological properties were also calculated (Table 4). The number of nodes and edges, clustering coefficient, and average degree were always highest in the water network, indicating that bacteria in water samples had the highest number of co-occurrences. The edge and node numbers in air networks were the least, for which the average path length was also the lowest, suggesting that bacteria in air samples were less connected but those correlated with each other were quite closely connected. Networks with small path lengths are considered to have “small-world” properties that are believed to be associated with the quick response of an ecosystem to perturbations (Watts and Strogatz, 1998; Zhou et al., 2011). This implies that bacteria in cave air maintains a small core tribe capable of strong resistance to environmental changes, a finding which deserves to be studied further in future works.

**TABLE 4:** Topological properties of co-occurring networks obtained from different karst cave niches.

**Cave niches**	**Nodes**	**Edges**	**Modularity (MD)**	**Clustering coefficient (CC)**	**Average path length (APL)**	**Network diameter (ND)**	**Average degree (AD)**
Air	138	244	0.647	0.307	4.832	13	3.536
Rock	193	805	0.510	0.445	5.456	17	8.342
Sediment	186	751	0.428	0.501	5.024	17	8.075
Water	223	1744	0.406	0.525	5.226	16	15.641

## Discussion

Though caves are generally considered as being extreme subterranean environments, in that they have limited availability of organic matter and light, have high humidity, and vary in their mineral composition, diverse bacteria can nonetheless thrive in these habitats (Northup and Lavoie, 2001). This empirical study performed an intensive analysis of bacterial communities dwelling in air, rock, sediment and water sampled from eight karst caves in southwest China. It represents an immense attempt to systematically document the pristine oligotrophic cave-associated bacteriomes. The discovery of a great number of bacteria with multiplex phylogenetic assignments will spur further investigation of microbe-involved biogeochemical processes in cave systems.

### Distribution Pattern of the Karst Cave Bacteriome

All caves studied in this work are rarely accessible to tourists, so any anthropogenic impact could only come from local residents occasionally entering the caves. Besides regular nutrient sources carried in via dripping water or flooding, bat roosts were also found in the sampled caves. These caves were geographically isolated overall, yet we found that the diversity and community composition were similar among the eight caves, with the *Proteobacteria*, *Actinobacteria*, *Firmicutes*, *Thaumarchaeota*, *Gemmatimonadetes*, *Chloroflexi*, and *Bacteroidetes* being the most abundant taxa found. Similar results were reported for caves located in Spain, Czech Republic, and Slovenia (Schabereiter-Gurtner et al., 2004; Porca et al., 2012). All the dominant phyla identified in this study were also found in previous studies, as summarized by Hershey and Barton (2018).

However, we did find significant differences among bacterial communities in terms of their occupied niches (i.e., air, rock, sediment, and water), not unlike Brannen-Donnelly and Engel (2015) who reported that bacterial community composition in cave streams and sediments differed at the class level. More recently, Alonso et al. (2018) reported that mineral substrate was the most significant factor determining cave microbial diversity and structure. Other studies also showed that the bacterial communities associated with dripping water in the Heshang Cave in central China were mostly dominated by *Proteobacteria*, while *Actinobacteria* was the most dominant phyla in weathered rocks in the same cave (Yun et al., 2016a, b). Those findings coupled with our results, suggest that specific niches drive microbial community evolution, perhaps sustaining the enrichment of unique microbial populations, despite the general similarity of the microbial community across whole caves.

### Niches (Water, Air, Sediment, Rock) Enrich Specific Bacterial Groups

Water samples in this study were collected from the cave ground surface, unlike studies that focused on bacteria in dripping water (e.g., Marques et al., 2019). In previous studies, *Betaproteobacteria* represented the core microbial groups occurring in karst water (Shabarova and Pernthaler, 2010; Shabarova et al., 2013; Brannen-Donnelly and Engel, 2015). Based on the latest SILVA database release 132, we found that the *Betaproteobacteriales*, *Pseudomonadales*, *Pseudonocardiales*, and *Bacillales* comprised a larger proportion of the community based on their average abundances in the cave water samples.

Bacteria dispersal in cave air is a poorly understood process. Recently, Leplat et al. (2019) monitored aerial rates of bacteria in five decorated caves for more than 2 years, yet no linear or quadratic relation was found between the distance index and the bacterial rates. In our study, the influence of sampling depth – the distance from cave entrance to sampling site – on bacterial community was also evaluated, but no statistically significant effect of sampling depth on either alpha or beta diversity was evident. We did, however, notice that genera in *Enterobacteriaceae* and *Staphylococcus* were identified as indictor genera of air samples, and such an observation might be related to bat residency or human activity in caves (Griffin et al., 2014). Discerning the impact of human activity on cave bacterial community can be complicated. For example, Hunter et al. (2004) identified coliforms, an indicator of fecal contamination in the Lechuguilla Cave, while Johnston et al. (2012) proved that a human urine-impacted site in a cave was colonized by endemic cave species instead of human commensal organisms.

Previous studies have reported that *Actinobacteria* represents the most abundant member in cave sediment communities (De Mandal et al., 2015a, b), however, we found that *Proteobacteria* also accounted for a large number of cave-dwelling bacteria. Consistent with this result, *Proteobacteria*, especially *Gammaproteobacteria*, were also prevalent in sediment samples from other caves (Barron et al., 2010; Brannen-Donnelly and Engel, 2015). Furthermore, Carmichael et al. (2013) found that cave sediments contained primarily *Proteobacteria* when they had been influenced by water influx. In our caves, multiple factors clearly affected the community composition of their sediment samples, with moisture being the most important one (Table 3, *R*^2^ = 0.4577, *p* = 0.001). Moisture also had a relatively strong positive association with the Shannon index in sediment samples, a result probably explained by water content being a major limitation for nutrient diffusion (Skopp et al., 1990). Interestingly, total nitrogen had a relatively strong negative relationship (Table 3, ρ = –0.5122, *p* = 0.001) with the Shannon index in sediment samples. It was reported recently that microbes in karst sediments are more limited by carbon and phosphorus than by nitrogen (Chen et al., 2019); so, it could be presumed that nitrogen sources in cave sediment may favorably enrich certain dominant taxa, thus decreasing overall species richness in the cave bacterial community.

Several studies have revealed that *Proteobacteria* can dominate cave rock ecosystems (Pasić et al., 2010; Engel et al., 2013; Ivanova et al., 2013; Gulecal-Pektas, 2016). We found that *Actinobacteria* was mostly predominant. Our results might be justified for two reasons: (1) Some of the aforementioned caves are so-called show-caves, with pre-historic paintings on their wall, whose increased availability of nutrients may lead to the dominance of *Proteobacteria*, as proposed by Tomczyk-Żak and Zielenkiewicz (2016). (2) Previous studies mainly focus on the surface of cave rock (Pasić et al., 2010; Engel et al., 2013; Ivanova et al., 2013; Gulecal-Pektas, 2016), while in our study, the surface of rock samples was first washed and attention was instead focused on bacteria living inside the rock or tightly attached to it. Remarkably, *Pseudonocardia* was identified as an indicator genus of rock bacterial community in our study. In prior studies, *Pseudonocardia* was deemed a major constituent of microbial colonization on walls with Paleolithic paintings in caves (Stomeo et al., 2008, 2009) and other subterranean niches such as provided by tombs (Diaz-Herraiz et al., 2013). Together, these findings suggest that microbial outbreaks in show caves might arise from an enrichment of cave indigenous bacteria instead of extraneous ones. Consistent with those studies, *Pseudonocardia* was abundant in our karst caves, where it served as a reliable indicator of the rock bacterial community.

### Keystone Members in the Cave Bacteriome

Association networks can provide a highly compressed and simplified version of the typically complex ecological interactions that shape bacterial communities (Eiler et al., 2011). Previous studies demonstrated that nodes with high betweenness centrality (BC) scores were particularly important for maintaining the connectivity of an ecological network (González et al., 2010). *Nitrosococcaceae* wb1-P19 (average abundances: air, 0.32%; rock, 4.42%; sediment, 6.15%; water, 5.20%), the uncultured *Rokubacteriales* member (average abundances: air, 0.02%; rock, 0.14%; sediment, 0.87%; water, 0.32%), and the uncultured *Gaiellales* member (average abundances: air, 0.17%; rock, 1.96%; sediment, 2.57%; water, 1.40%) were identified as the top three in terms of their BC scores in this study. We therefore hypothesize that they might play critical roles in maintaining the structure and function of karst cave communities. The wb1-P19 is a genus named after an uncultured clone obtained from cave water, and it phylogenetically clustered with a section of sulfur- or nitrite-oxidizing autotrophic bacteria (Holmes et al., 2001). *Rokubacteriales* belongs to *Candidatus Rokubacteria*, whose genomes are large, with high G+C content and the potential for nitrogen respiration (Becraft et al., 2017). *Gaiellales* is a deep-branching lineage of *Actinobacteria*, and its metabolic potential is only represented by a single cultured species, *Gaiella occulta*; it is predicted to reduce nitrate and to perform CO_2_ fixation (Severino et al., 2019). Members of *Gaiellales* were also found in other subterranean environment such as volcanic caves (Riquelme et al., 2015), supporting their general existence in dark cave systems.

## Conclusion

This study provides a comprehensive assessment of the bacterial communities associated with eight karst caves in southwest China. Taking species richness and composition into account, the cave bacteriome shows similarities among the caves investigated but differs significantly among different cave niches, and different environment factors drive niche-specific bacterial assembling. Indicator genera are predicted to reflect the unique environment of each cave niche. A co-occurrence analysis suggests that cave bacterial community is well modularized, and that the identified keystone genera might play a critical role in carbon and nitrogen, as well as sulfur, biogeochemical cycles, thus providing a new perspective on microbial assembly in cave ecosystems. Networks of the four types of samples (corresponding to four niches for bacterial taxa) showed different topological properties, arising from the distinct co-occurrence patterning of bacterial communities in each cave niche.

## Data Availability

Raw sequence reads of the 16S rRNA gene amplicon were deposited in the NCBI Sequence Read Archive (SRA) under project accession number PRJNA497480.

## Author Contributions

H-ZZ carried out the experiments, analyzed the data, and wrote the manuscript. Z-FZ collected the samples and performed the experiments. NZ and B-JW conducted part of the lab work. C-YJ, LC, and S-JL designed the experimental plan. S-JL polished the manuscript.

## Conflict of Interest Statement

The authors declare that the research was conducted in the absence of any commercial or financial relationships that could be construed as a potential conflict of interest.

## Footnotes

1 http://whc.unesco.org/en/list/1248/

2 https://www.R-project.org/

3 https://github.com/droglenc/FSA

4 https://CRAN.R-project.org/package=vegan

5 http://biostat.mc.vanderbilt.edu/wiki/Main/Hmisc
