# Supplementary material for: Diversity, Distribution and Co-occurrence Patterns of Bacterial Communities in a Karst Cave System
Source: Front Microbiol. 2019 Aug 6;10:1726. doi: 10.3389/fmicb.2019.01726 (PMC6691740; doi:10.3389/fmicb.2019.01726)
Supplement: Supplementary file 1 [file Table_1.DOCX]

**Supplementary Material**

**Supplementary Table 1** Statistics of samples from different caves and different niches

| **Cave** | **Air** | **Rock** | **Soil** | **Water** | **Total** |
| --- | --- | --- | --- | --- | --- |
| **C1** (N 29º35´6.09"/E 108º0´2.28") | 6 | 6 | 6 | 3 | **21** |
| **C2** (N 29º35´27.00"/E 108º0´2.00") | 4 | 5 | 6 | 3 | **18** |
| **G1** (N 24º56´30.55"/E 110º30´37.93") | 5 | 5 | 5 | 1 | **16** |
| **G3** (N 23º24´36.00"/E 108º55´52.00") | 4 | 4 | 4 | 2 | **14** |
| **S7** (N 28º11´24.60"/E 105º8´21.60") | 5 | 5 | 4 | 3 | **17** |
| **S8** (N 30º24´36.00"/E 106º52´42.00") | 4 | 4 | 4 | 2 | **14** |
| **Y2** (N 25º8´4.00"/E 103º22´57.00") | 5 | 5 | 5 | 3 | **18** |
| **Y3** (N 24º28´11.52"/E 102º50´53.00") | 3 | 2 | 3 | 2 | **10** |
| **Total** | **36** | **36** | **37** | **19** | **128** |
