# Supplementary material for: Diversity, Distribution and Co-occurrence Patterns of Bacterial Communities in a Karst Cave System
Source: Front Microbiol. 2019 Aug 6;10:1726. doi: 10.3389/fmicb.2019.01726 (PMC6691740; doi:10.3389/fmicb.2019.01726)
Supplement: Supplementary file 4 [file Table_4.DOCX]

**Supplementary Material**

**Supplementary Table 4** Pairwise evaluation of differences between bacterial communities in different cave niches

| **Pairs** | **F Model** | **R^2^** | **adjusted *p* value** |
| --- | --- | --- | --- |
| Air vs Rock | 8.08395 | 0.103529 | 0.006** |
| Air vs Soil | 10.15242 | 0.125103 | 0.006** |
| Air vs Water | 5.16194 | 0.088751 | 0.006** |
| Rock vs Soil | 2.337844 | 0.031878 | 0.012* |
| Rock vs Water | 1.837273 | 0.033504 | 0.006** |
| Soil vs Water | 1.360881 | 0.024582 | 0.3 |
