# Supplementary material for: Diversity, Distribution and Co-occurrence Patterns of Bacterial Communities in a Karst Cave System
Source: Front Microbiol. 2019 Aug 6;10:1726. doi: 10.3389/fmicb.2019.01726 (PMC6691740; doi:10.3389/fmicb.2019.01726)
Supplement: Supplementary file 6 [file Data_Sheet_1.docx]

**Supplementary Material**


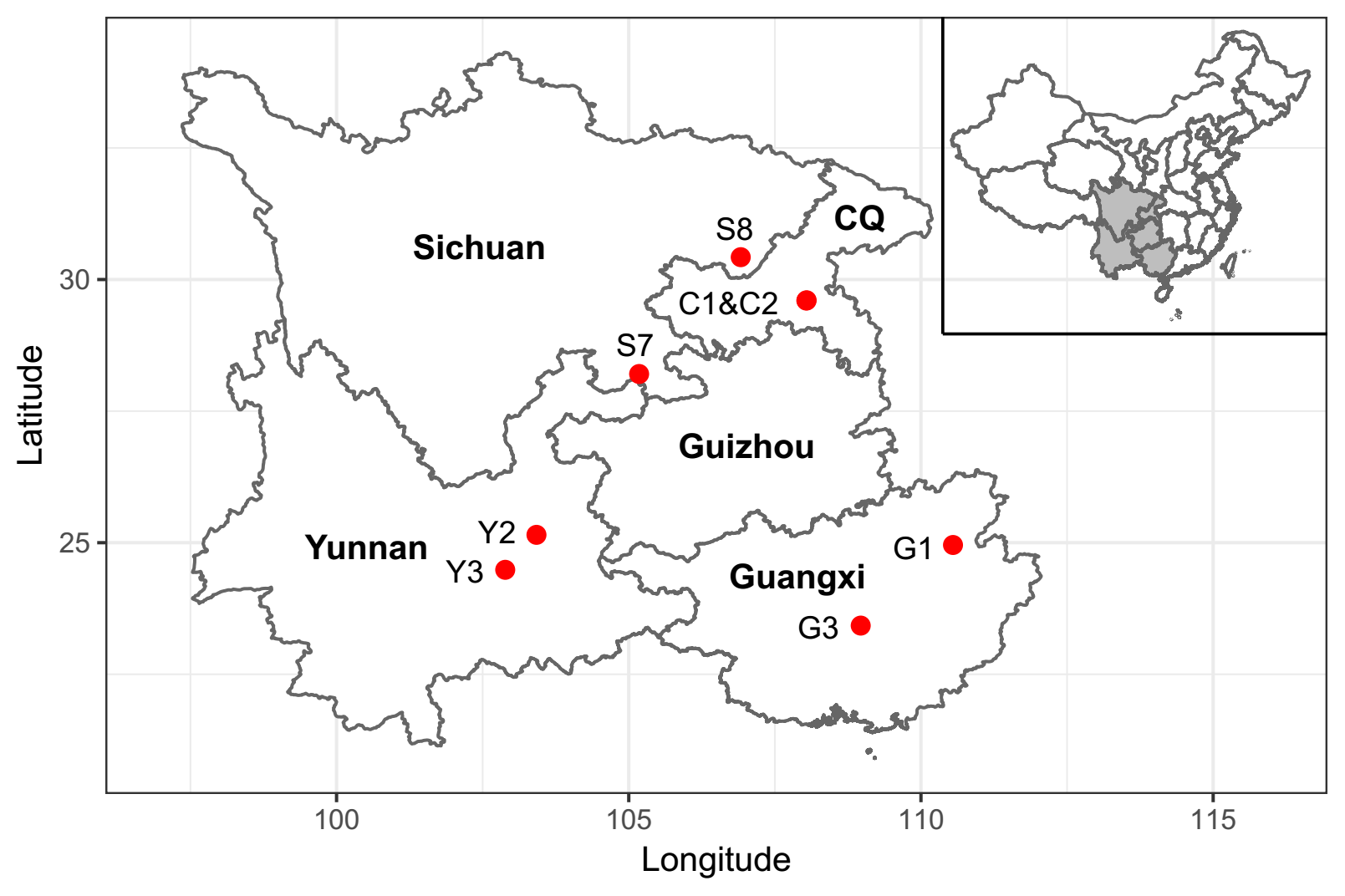


**Supplementary Figure 1.** Distribution of sampling caves. CQ is short for Chongqing, C1 & C2 are two closely adjacent caves that can not be separately marked at the shown scale.


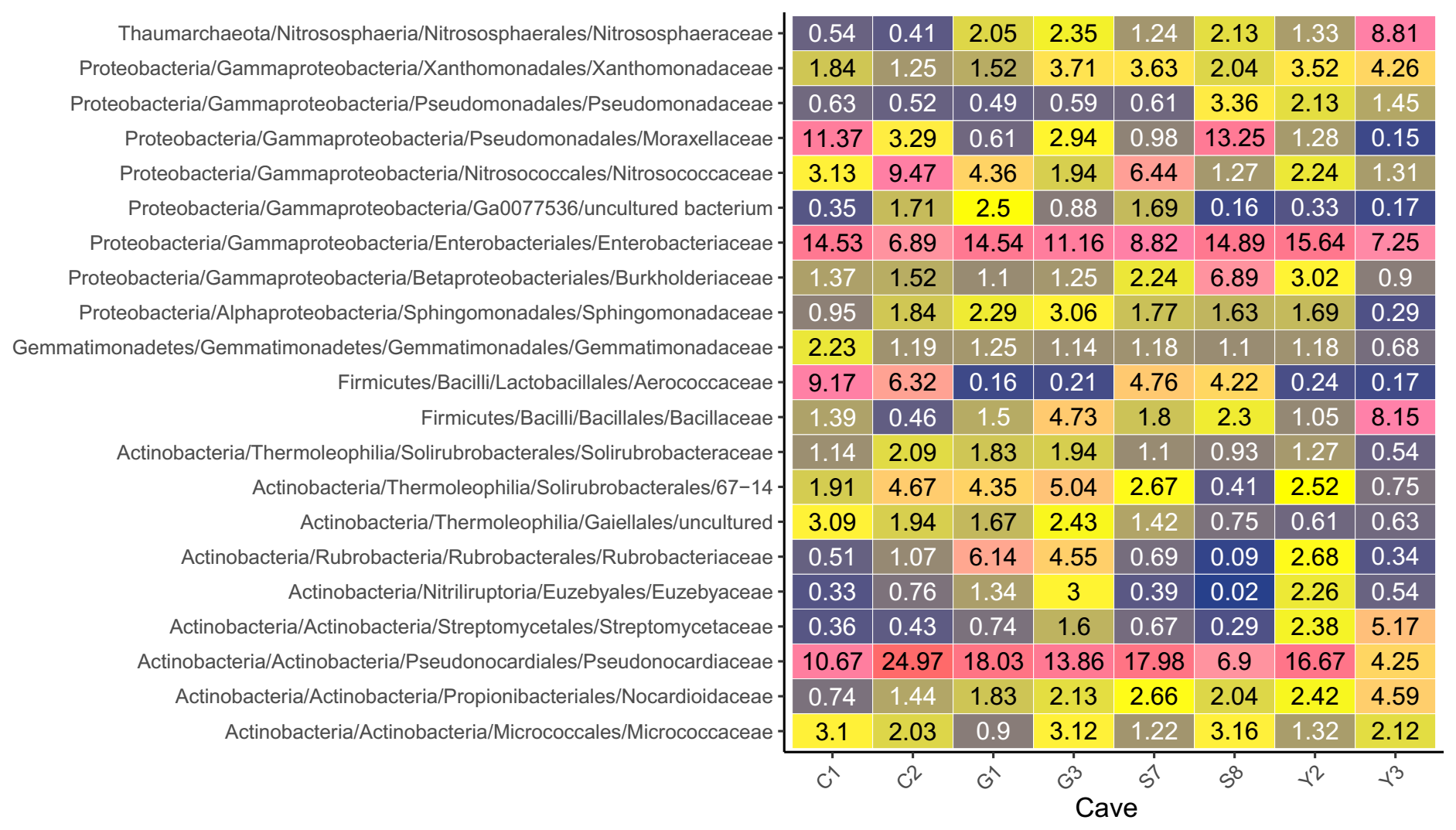


**Supplementary Figure 2.** The average relative abundance of abundant cave bacterial family in each sampled cave. The number within each box represents the median abundance (%) of the taxa in the certain cave. Taxa that cannot be classified to family level are labeled as “Unclassified”. Taxa with low abundance are colored blue, higher abundance are yellow, and the highest are in pink.


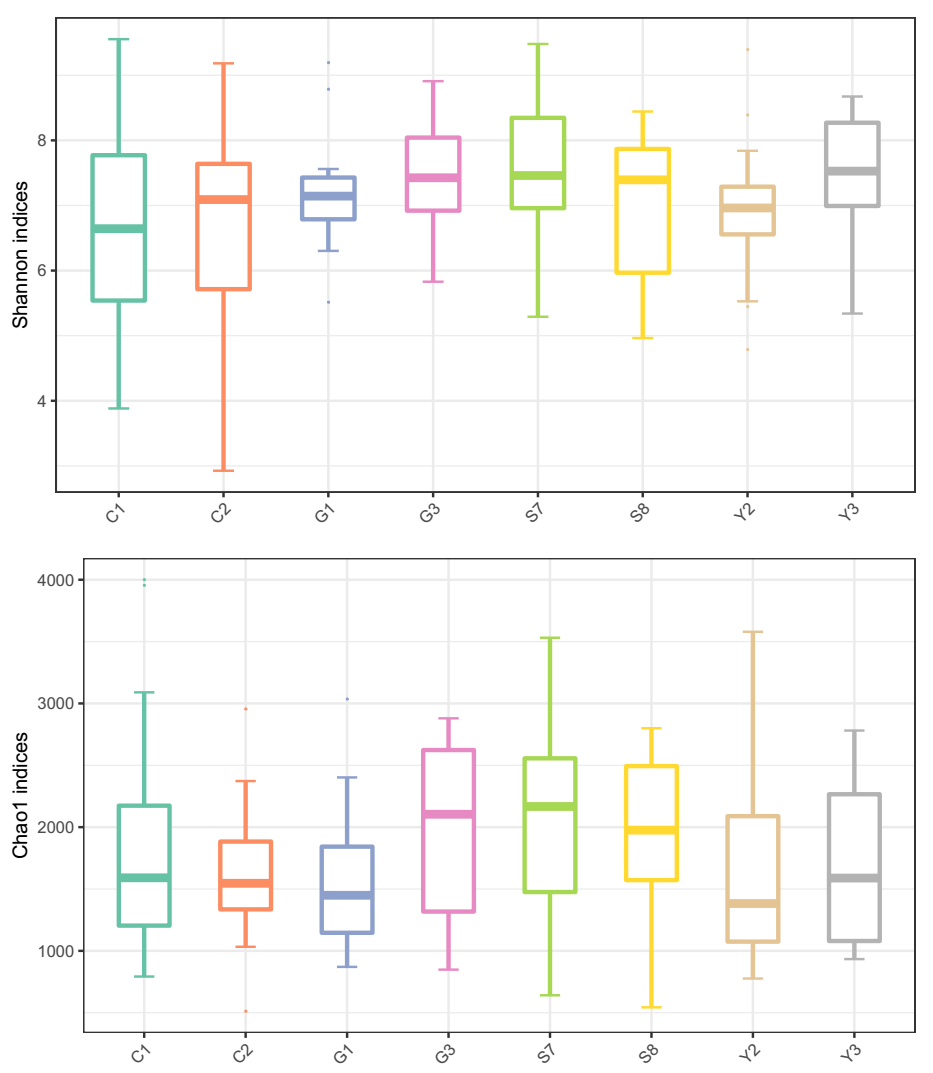


**Supplementary Figure 3.** Alpha diversity of bacterial communities in different caves measured by Shannon and Chao1 indices.


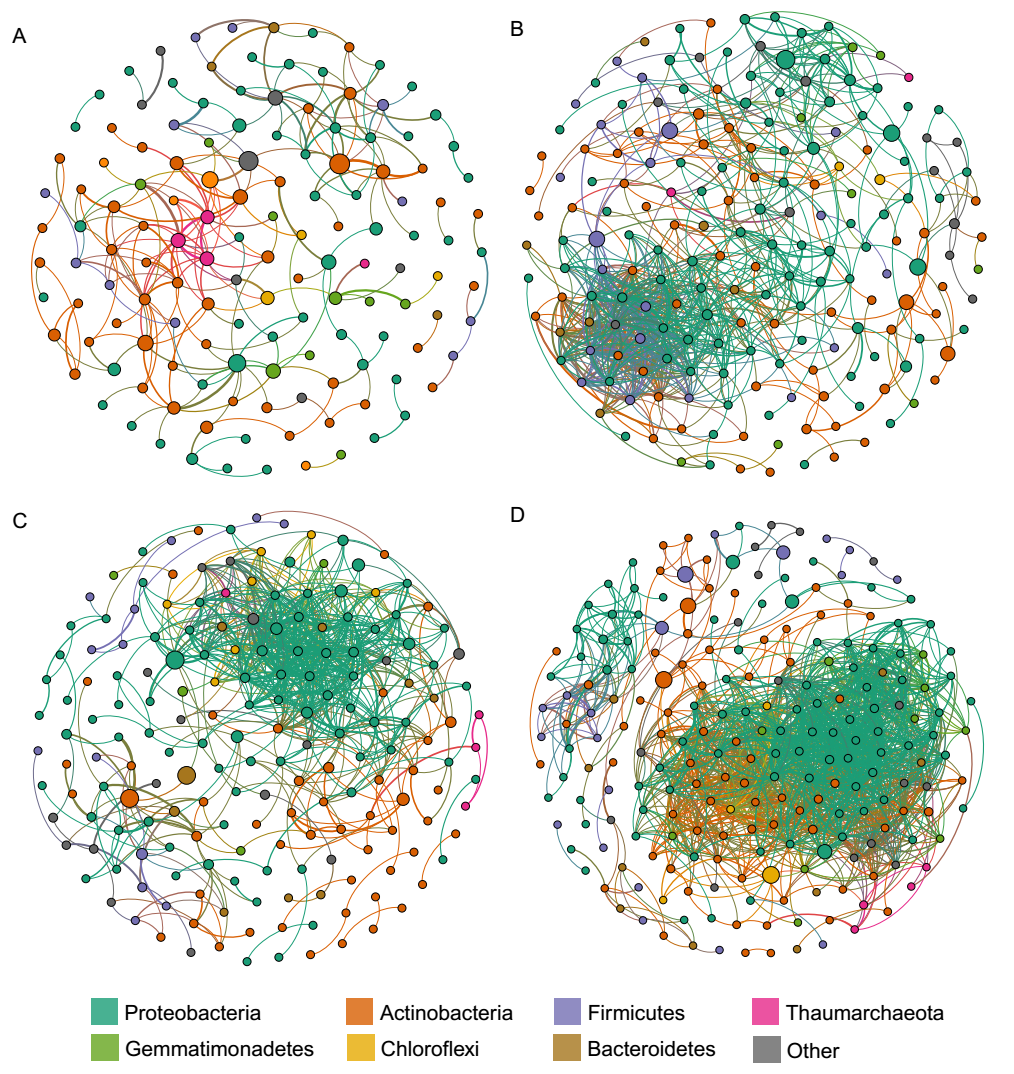


**Supplementary Figure 4.** Network of co-occurring microbial genera based on correlation analysis for air (A), rock (B), sediment (C) and water (D) samples. A connection stands for a strong (Spearman’s ρ > 0.6) and significant (*p* < 0.01) correlation. The size of each node is proportional to the relative abundance, the nodes were colored by phylum.
